# Combination of Percutaneous Endoscopic Gastrostomy and Lumboperitoneal Shunt: A Case Report

**DOI:** 10.7759/cureus.70527

**Published:** 2024-09-30

**Authors:** Tatsuya Tanaka, Takahiro Kumono, Tomoyuki Naito, Fumitaka Yamane, Akira Matsuno

**Affiliations:** 1 Department of Neurosurgery, International University of Health and Welfare Narita Hospital, Narita, JPN; 2 Department of Neurosurgery, International University of Health and Welfare Atami Hospital, Atami, JPN

**Keywords:** gastrostomy, hydrocephalus, infection, lateral approach, lumboperitoneal shunt, percutaneous endoscopic gastrostomy

## Abstract

The combination of percutaneous endoscopic gastrostomy (PEG) and cerebrospinal fluid (CSF) shunt surgery presents unique challenges in managing shunt-related infections. Although the association between PEG and ventriculoperitoneal (VP) shunt surgery is well documented, studies on the combination of PEG and lumboperitoneal (LP) shunt surgeries are limited.

We report the case of a 70-year-old man who developed hydrocephalus after decompressive craniectomy for ischemic stroke. The patient required PEG for nutritional support and an LP shunt for CSF drainage. PEG was initially performed. After 16 days, an LP shunt was placed using the lateral approach to maximize the distance between the PEG site and abdominal incision. Subcutaneous CSF leakage was resolved without any shunt infection at the three-month follow-up.

This case highlights the importance of strategically combining PEG and LP shunts to minimize infection risk. Maximizing the distance between the PEG site and abdominal incision for the LP shunt may help prevent shunt-related infections, warranting further clinical investigation.

## Introduction

Cerebrospinal fluid (CSF) shunt procedures are commonly performed in patients with hydrocephalus secondary to head trauma or cerebrovascular accident. When patients require long-term nutritional support, percutaneous endoscopic gastrostomy (PEG) is often preferred as a means of artificial nutrition [1]. However, when PEG is combined with CSF shunt procedures, the risk of shunt infection becomes a significant concern, thereby complicating management [2-8]. Although many reports have addressed the combination of PEG and ventriculoperitoneal (VP) shunting [2-7], only a few studies have discussed the combination of PEG and lumboperitoneal (LP) shunting [8]; thus, the infection rates and management strategies remain unclear. Here, we report the case of a patient who underwent both PEG and LP shunting, discuss the management approach, and review the relevant literature.

## Case presentation

**Figure 1:**
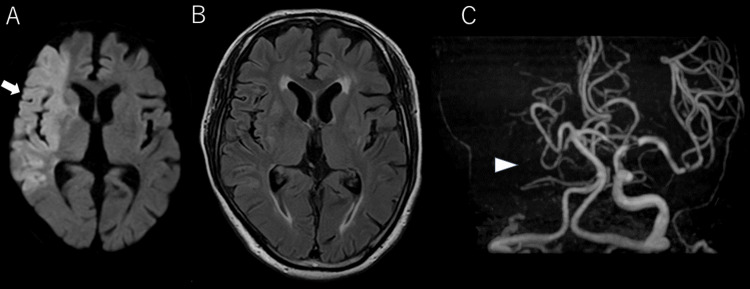
Initial MRI and MRA (A) Initial brain MRI diffusion-weighted images showing brain infarction in the right middle cerebral artery territory (arrow). (B) Fluid-attenuated inversion recovery images showing no ischemic changes. (C) MRA indicating occlusion of the right internal carotid artery (arrowhead). MRI, magnetic resonance imaging; MRA, magnetic resonance angiography

A 70-year-old man with a history of hypertension and bladder cancer was found vomiting and unable to move inside his car. The patient was transported to our hospital by ambulance. Upon arrival at the hospital, the patient’s vital signs were as follows: heart rate 79 beats/min (irregular). blood pressure 180/97 mmHg, respiratory rate 20 breaths/min, SpO2 92% at room air, temperature 36.3°C, and Glasgow Coma Scale (GCS) score 14 (E4V4M6). The patient exhibited left-sided hemiparesis and left-sided neglect, with a National Institutes of Health Stroke Scale score of 16. Diffusion-weighted imaging (DWI) revealed hyperintensity in the right middle cerebral artery (MCA) territory; however, no ischemic changes were observed on fluid-attenuated inversion recovery (Figures 1A, 1B). The ASPECTS DWI score was 4. Magnetic resonance angiography confirmed right internal carotid artery occlusion (Figure 1C). Electrocardiography revealed no atrial fibrillation.

**Figure 2:**
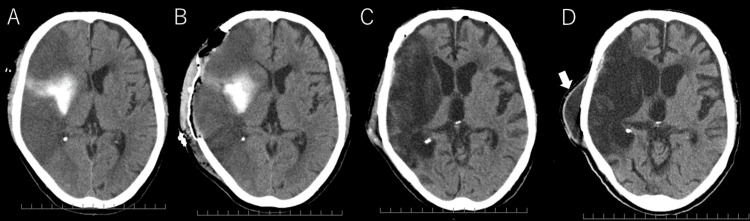
Head CT changes over time (A) CT scan on day 2 showing extensive cerebral infarction in the right middle cerebral artery territory, ventricular deformation, and midline shift. (B) Postoperative CT scan showing the status after decompressive craniectomy. (C) CT on day 117 revealing post-cranioplasty. (D) Enlarged ventricles and subcutaneous cerebrospinal fluid collection (arrow) are also noted. CT, computed tomography

The patient was diagnosed with ischemic stroke due to large vessel occlusion with an unknown onset time and underwent thrombectomy, which failed to achieve recanalization. On the second day of admission, the patient’s GCS score decreased to 10 (E3V3M4), and his right pupil became dilated. Computed tomography (CT) revealed low attenuation in the right MCA territory, ventricular deformation, and midline shift; thus, the patient was diagnosed with brain herniation (Figure 2A). Decompressive craniectomy was performed (Figure 2B). On day 88, cranioplasty was performed (Figure 2C), and on day 117, the patient was transferred to a rehabilitation hospital with a GCS score of 13, left hemiparesis, nasogastric tube feeding, and a modified Rankin Scale score of 5.

**Figure 3:**
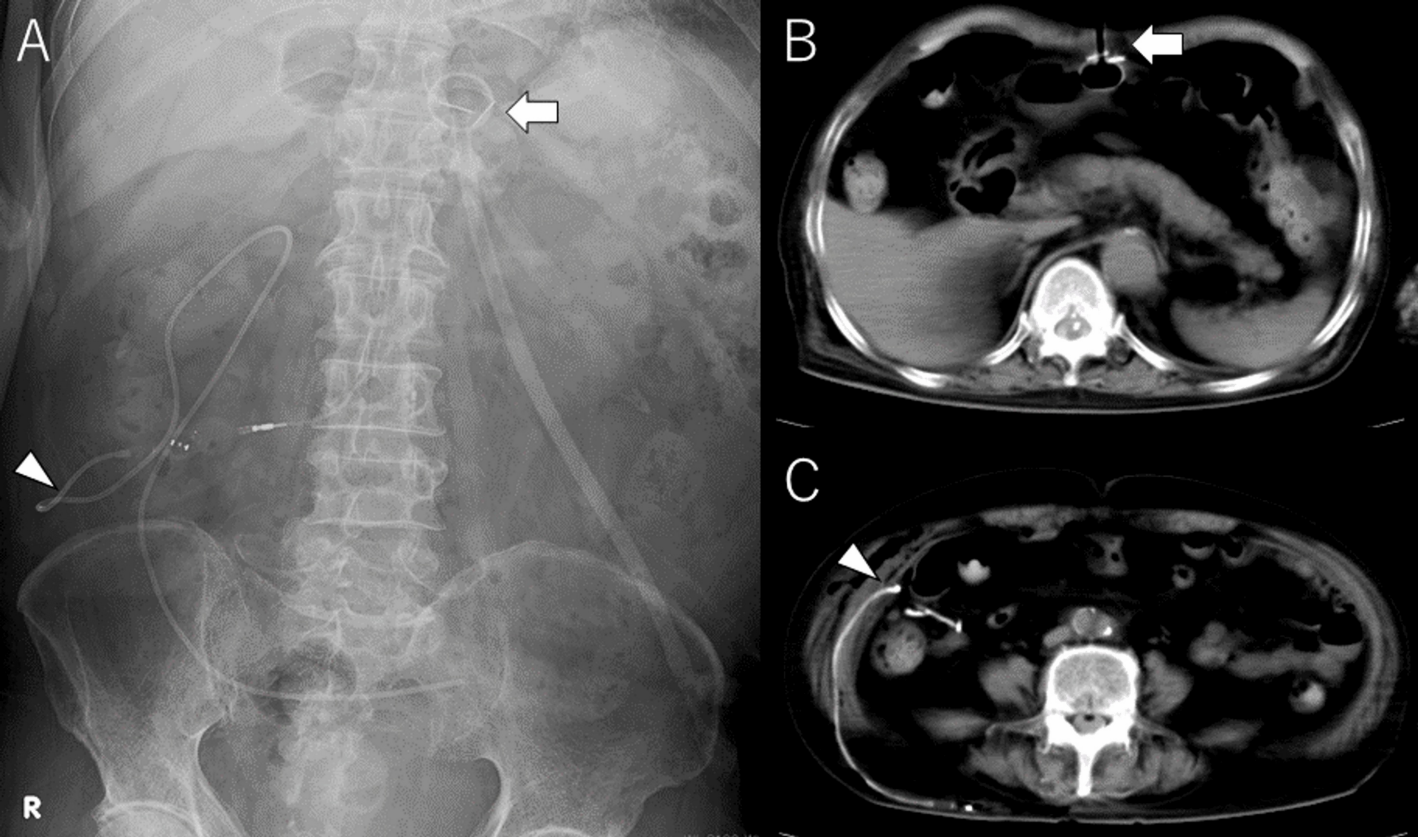
Images after PEG and LP shunt (A) Abdominal X-ray showing the position of the PEG tube (arrow) and the LP shunt insertion site (arrowhead). (B, C) Abdominal computed tomography after PEG and LP shunt placement showing the PEG tube in the upper abdomen (arrow) and the distal catheter passing between the internal oblique and transversus abdominis muscles, inserted into the right lower abdomen (arrowhead). PEG, percutaneous endoscopic gastrostomy; LP, lumboperitoneal

Subcutaneous fluid collection was observed at the right craniotomy site. Head CT revealed subcutaneous fluid accumulation and ventricular enlargement (Figure 2D). The patient was diagnosed with subcutaneous CSF leakage due to hydrocephalus that developed after right-sided cranioplasty. Despite head elevation and CSF drainage via lumbar puncture, the patient’s condition did not improve, and LP shunting was planned. Furthermore, due to the persistent difficulty with oral intake, PEG was also planned. It was decided to perform PEG first, followed by LP shunt placement. On day 176, PEG was performed, and the optimal site was identified by observing the indentation sign from within the gastric lumen via endoscopy. Gastrostomy was created in the upper abdomen using the PULL technique, and cefazolin 1 g was administered once preoperatively and once postoperatively. The gastrostomy tract was confirmed to be free of infection (Figures 3A, 3B). On day 192, an LP shunt (Codman CERTAS Plus adjustable shunt valve with SiphonGuard, Integra Life Sciences, Princeton, NJ, USA) was placed. Under general anesthesia, a skin incision was made in the right lower abdomen at the L4 level. The external oblique, internal oblique, and transversus abdominis muscles were bluntly dissected, and the peritoneum was incised to access the peritoneal cavity. The shunt passer was navigated through the muscle layers using finger guidance, and the distal catheter was inserted into the peritoneal cavity (Figures 3A, 3C). The distance between the PEG insertion site and the LP shunt incision site was 32 cm (Figure 3B). Cefazolin 1 g was administered once preoperatively and once postoperatively.

**Figure 4:**
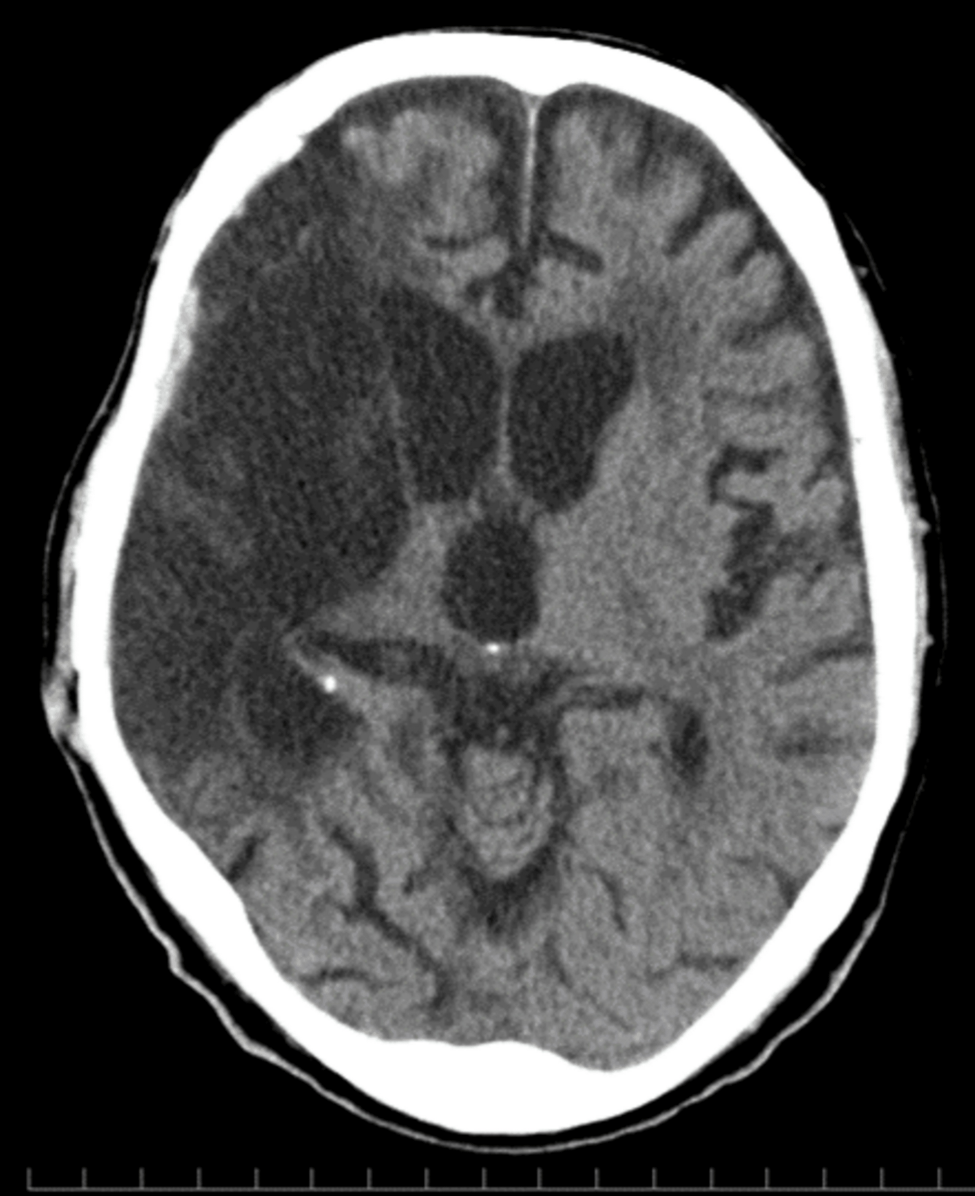
CT after LP shunt CT of the post-LP shunt showing the resolution of the subcutaneous CSF collection. CT, Computed tomography; LP, lumboperitoneal

The shunt valve pressure was initially set at a pressure setting of 7 and gradually reduced to 5. Subcutaneous CSF leakage resolved within two weeks postoperatively (Figure 4), and no shunt infection was observed at the three-month follow-up. The patient is currently receiving a combination of oral intake and PEG nutrition.

## Discussion

This case involved the combination of PEG and LP shunting. Although both CSF shunt procedures for hydrocephalus following cerebrovascular accident and PEG for feeding difficulties are frequently performed, reports on the combination of PEG and VP shunt are more common [2-7]. In contrast, reports on the combination of PEG and LP shunting are limited [8]. A PubMed search using the terms “Lumboperitoneal shunt,” “Percutaneous endoscopic gastrostomy,” and “Gastrostomy” yielded no relevant reports on the combination of PEG and LP shunting, with only one case series found in the Japan Medical Abstracts Society database.

Regarding VP shunts, a recent systematic review reported a median shunt infection rate of 5.1% (range: 0%-50%) and an estimated pooled prevalence of 7.41% among patients who underwent VP shunt placement combined with PEG [2]. The infection risk was found to be twice as high in these patients compared with that in those who did not require PEG [2]. Most bacteria causing VP shunt infections are skin flora, and infections are most likely to occur during the surgery or postoperative wound healing period [2]. Therefore, the insertion of a PEG tube may increase the risk of shunt infection in patients with VP shunts [2-7]. Maintaining sufficient distance between the PEG site and the abdominal shunt catheter site is considered necessary [6]. The location of the PEG site is generally fixed because it is chosen based on the proximity of the abdominal wall to the gastric wall. However, the insertion site of the shunt catheter is adjustable, and placement in the right lower abdomen is recommended [8]. In idiopathic normal-pressure hydrocephalus, LP shunting has been previously reported to be as effective as VP shunting [9], and its use is expected to increase because of its less invasive nature concerning the brain. Regarding the combination of PEG and LP shunting, Tanaka et al. reported an infection rate of one (6.25%) of 16 cases, and the longer distance between the PEG and abdominal incision sites was suggested to contribute to the lower infection rate [8]. Regarding the method of distal catheter placement in LP shunting, the lateral approach, in which the catheter is passed through the muscle layers of the external and internal oblique muscles, has been previously reported [10,11]. In the patient in this case report, to prevent infection, the catheter was inserted via the lateral approach, ensuring a longer distance between the PEG site and the abdominal incision site.

LP shunting was performed 16 days after PEG placement to confirm the absence of skin infection. Previous reports have suggested that the sequence and interval between PEG and VP shunt placement influence the risk of infection [3-7]. However, the aforementioned systematic review found no difference in infection risk related to the order of insertion of the VP shunt and PEG [2]. The placement of a PEG tube is a risk factor for VP shunt infection; however, the infection risk did not increase with the interval, regardless of the order of insertion of the VP shunt and PEG [2]. Similarly, in the combination of PEG and LP shunting, it is believed that the order and interval between placements do not correlate with shunt infection and that good healing and the absence of infection at each surgical site are critical for preventing shunt infection.

Prophylactic antibiotics have been shown to reduce the risk of infection in CSF shunt surgery, regardless of shunt type, patient age, or antibiotic duration [9,12,13]. Extending antibiotic use beyond 24 hours has not shown additional benefits [9,12,13]. Therefore, extended antibiotic administration is not recommended in the combined procedure of CSF shunt and PEG.

The limitation of this case report is that reports on the combination of PEG and LP shunting are extremely limited, and overall evidence is scarce. Therefore, further research on the combination of LP shunting and PEG is necessary.

## Conclusions

This case report discusses the combination of LP shunting for hydrocephalus following cerebrovascular accident and PEG for feeding difficulties, with a focus on postoperative management. In this case, LP shunting was performed after confirming adequate wound healing after PEG placement. The lateral approach for distal catheter insertion may play a crucial role in reducing infection risk by ensuring a greater distance between the PEG site and the abdominal incision site. However, studies on the combination of PEG and LP shunting are extremely limited, highlighting the need for further case accumulation and research.
